# Sub-Second Dopamine Detection in Human Striatum

**DOI:** 10.1371/journal.pone.0023291

**Published:** 2011-08-04

**Authors:** Kenneth T. Kishida, Stefan G. Sandberg, Terry Lohrenz, Youssef G. Comair, Ignacio Sáez, Paul E. M. Phillips, P. Read Montague

**Affiliations:** 1 Human Neuroimaging Laboratory, Virginia Tech Carilion Research Institute, Roanoke, Virginia, United States of America; 2 Departments of Psychiatry & Behavioral Sciences and Pharmacology, University of Washington, Seattle, Washington, United States of America; 3 Department of Neuroscience, Baylor College of Medicine, Houston, Texas, United States of America; 4 Department of Surgery, Division of Neurosurgery, American University of Beirut, Lebanon; 5 Department of Physics, Virginia Polytechnic Institute and State University, Blacksburg, Virginia, United States of America; 6 The Wellcome Trust Centre for Neuroimaging, University College London, United Kingdom; University of Missouri, United States of America

## Abstract

Fast-scan cyclic voltammetry at carbon fiber microelectrodes allows rapid (sub-second) measurements of dopamine release in behaving animals. Herein, we report the modification of existing technology and demonstrate the feasibility of making sub-second measurements of dopamine release in the caudate nucleus of a human subject during brain surgery. First, we describe the modification of our electrodes that allow for measurements to be made in a human brain. Next, we demonstrate *in vitro* and *in vivo*, that our modified electrodes can measure stimulated dopamine release in a rat brain equivalently to previously determined rodent electrodes. Finally, we demonstrate acute measurements of dopamine release in the caudate of a human patient during DBS electrode implantation surgery. The data generated are highly amenable for future work investigating the relationship between dopamine levels and important decision variables in human decision-making tasks.

## Introduction

The neurotransmitter dopamine has been implicated in both motoric and cognitive functions, especially those associated with reward valuation processes [1]–[3]. Computational models of dopamine function have been validated at the level of single unit activity in non-human primates and rodents [4]–[7]. Recent advances in neurochemical monitoring have enabled sub-second dopamine detection in rodents during behavioral tasks [8], [9], providing the capacity for computational testing at the level of dopamine release [10]. Nevertheless, *in vivo* measurements of extracellular dopamine in human brains are currently restricted to timescales afforded by microdialysis [11] or imaging [12] methods that do not resolve sub-second computations mediated by dopamine release. However, the now routine surgical implantation of neuroprosthetic devices for deep-brain stimulation (DBS) in the treatment of Parkinson's disease [13] provides a window of opportunity for unprecedented monitoring of neurotransmission in the human brain using invasive electrode-based techniques [14]. Here, we describe the use of proven fast-scan cyclic voltammetry methods for detecting dopamine [8], [9] and the modification of existing biocompatible electrodes [15] for use in human brain; we demonstrate the feasibility of sub-second dopamine detection in human striatum, and discuss the potential of pairing this kind of measurement with human-decision making paradigms designed around current computational models of dopamine function [16]. This advance provides a mechanism for studying long-standing questions regarding dopamine's role in human cognition by permitting sub-second dopamine measurements in the human brain during behavioral tasks.

## Results and Discussion

**Figure 1:**
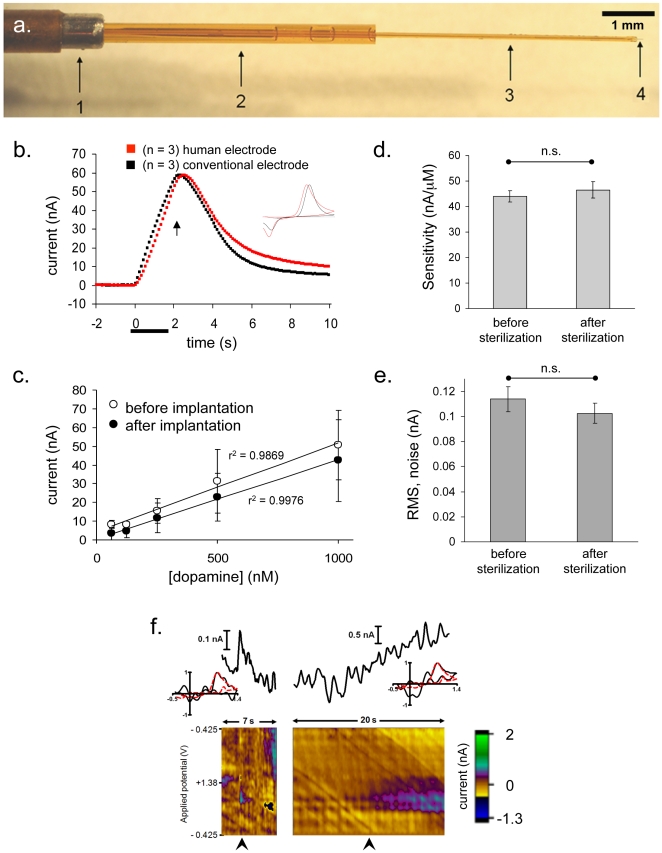
Human-compatible carbon fiber microelectrode can detect dopamine in human striatum. (**a**) Human electrode assembly comprised of protective tube with reference electrode at the tip (1), large-gauge fused silica capillary (2), small-gauge fused silica (3) and carbon fiber (4). (**b**) Average change in peak oxidative current in rat striatum following electrical stimulation (60 Hz, 120 pulses, 300 µA) of dopamine axon pathway *in vivo* recorded at conventional voltammetric electrodes or human electrode assembly (referenced to Ag/AgCl or stainless steel respectively; r^2^ = 0.934, p<0.0001, n = 3). The inset shows the average electrochemical signature at the maximum response (r^2^ = 0.887, p<0.0001). (**c**) Linear responses (vertical axis: peak oxidative currents for dopamine) of sterilized electrode responses to known concentrations of dopamine (62.5, 125, 250, 500 and 1000 nM) before (r^2^>0.987, p<0.001) and after (r^2^>0.997, p<0.0001) implantation in brain tissue. Data are mean ± SEM, n = 3. (**d**) Electrode sensitivity and (**e**) root-mean-square (RMS) noise is unchanged by gas sterilization with ethylene oxide (p>0.75 and p>0.65, paired t-test). Data are expressed as mean ± SEM, n = 7. (**f**) Voltammetric signals measured at electrode implanted in the patient's right caudate: (**left**) sub-second changes and (**right**) changes over several seconds. Changes in electrochemical current are measured at the peak oxidation potential for dopamine (+0.65–0.75 V). Insets show cyclic voltammograms from the patient's brain (black trace) compared to a standard reference cyclic voltammogram for dopamine (dashed red trace; r^2^ = 0.75 (left) and r^2^ = 0.765 (right), p<0.0001 for both). These cyclic voltammograms were measured at the time points indicated by the black arrowheads in the lower panels. The lower panels show two-dimensional plots with electrochemical current (nA, pseudo color, see color bar to the right) plotted against time (horizontal-axis) and applied potential (vertical-axis).

In developing instrumentation to detect dopamine in the human brain, we had three important criteria. The experimental methodology must be safe to the patient, compatible with existing neurosurgical apparatus and the operating-room environment, and capable of sub-second detection of physiological dopamine. To achieve these goals, we fabricated an electrode assembly for use with clinical stereotactic apparatus, based upon an existing biocompatible microsensor that has been validated for *in vivo* dopamine detection [15]. Intraoperative electrophysiology electrodes that are used for functional mapping during DBS-electrode implantation are 276.5-mm long, 360-µm in diameter and housed in a stainless-steel protective tube (Differential microTargeting™ electrode, FHC). Therefore, we constructed electrode assemblies to match these dimensions and housed them in identical protective tubes (Figure 1a; see Methods S1). First, a carbon fiber (7-µm diameter) was inserted into a fused silica tube (90-µm outer diameter, 20-mm long) and sealed as previously described [15]. This capillary was then fixed into a larger-diameter (360 µm) fused-silica capillary (271.5-mm long) leaving 5 mm exposed. Due to the relatively high electrical impedance of graphite (compared to metal wires), the carbon fiber is not suitable for conducting the signal along an electrode of this length while maintaining high-fidelity electrochemical recordings. Therefore, a silver wire within the larger capillary was used to bridge the back end of the capillary with the sensor, connecting to the carbon fiber with silver conductive paint. The electrode was then back loaded into a polyimide-coated, stainless-steel protective tube (FHC) which has 1 mm of stainless steel exposed at the tip for use as a reference electrode [17], [18]. Finally, a gold-plated pin connector was attached to the back end of the large capillary making electrical contact with the silver wire via conductive epoxy, and heat-shrink tubing was placed over the body of the connector, extending 5 mm along the fused-silica shaft.

The carbon-fiber sensor used in our electrode assembly is identical to that used for previous applications [9], [10], [15], thus shares the same electrochemical properties. Indeed, performance of this assembly (with stainless-steel reference) for *in vivo* detection of dopamine transmission was highly conserved relative to the conventional glass-insulated carbon-fiber recording electrode referenced to Ag/AgCl (n = 3 of each electrode type; Figure 1b). Specifically, detection of stimulated dopamine release in the rat produced responses that were similar in chemical identity (r^2^ = 0.89, p<0.0001; Figure 1b inset), temporal response (r^2^ = 0.95, p<0.0001; Figure 1b), and sensitivity (regression line slope = 0.981). Using fast-scan cyclic voltammetry, we tested the sensitivity of the electrode to dopamine *in vitro* before and after insertion in (rat) brain tissue for 30 minutes, a period approximating the anticipated implantation time in human brain. The electrochemical response to dopamine was linear with concentration and the sensitivity was not altered by brain implantation (Figure 1c). Importantly, for the microsensor to have utility for recordings in humans, it must be able to withstand sterilization. Gas sterilization with ethylene oxide did not alter the sensitivity or electrical noise level of the electrode (Figure 1d and 1e, respectively) indicating maintained integrity of the electrochemical surface and insulating seals.

We have developed and characterized an electrode assembly intended for use in humans. The assembly is biocompatible [15], can be sterilized without adverse effect on performance, and is housed in a protective tube that is routinely used for clinical neurosurgical applications and incorporates a reference electrode, thus eliminating the need for an additional implant. The electrode assembly shares the dimensions of an intraoperative neurophysiological electrode conferring compatibility with the stereotactic frame and manipulators used for the clinically-required neurosurgical procedures; and can be used with a laptop-based data-acquisition system that can be brought into an operating room. The microsensor shares the electrochemical properties of existing electrodes that are capable of detecting sub-second dopamine release in the nanomolar range [10], [15]. Together, these data demonstrate that our instrumentation is safe (see below), compatible with the neurosurgical environment and capable of sub-second dopamine detection at physiological levels, satisfying our criteria for a suitable methodology for *in vivo* neurochemical recording in humans.

Next, we tested our electrode in a single human subject using procedures approved by Baylor College of Medicine's Institutional Review Board. This proof-of-principle demonstration was conducted in a consenting patient (MH), who suffered from late-stage Parkinson's disease and was undergoing elective surgery for DBS-electrode implantation into the subthalamic nucleus. On the day of surgery, MH was not medicated with levodopa. Following routine intraoperative electrophysiology recording for functional mapping of the DBS target site, the carbon-fiber microelectrode assembly was placed in MH's right caudate nucleus at x = 19.18 mm, y = 9.13 mm, and z = 18.28 mm from the mid-commissural point. This target was afforded due to the proximity of the target location to the trajectory of the planned DBS electrode insertion path. During insertion, the carbon fiber electrode was retracted in the protective tube and advanced into the brain for final placement. The subject then engaged in a behavioral task [16] (see Methods S1) where the current value and recent history of a stock market was graphically represented on a laptop monitor. The subject chose the proportion of a portfolio (initially valued at $100) to be invested in the stock market. These decisions were submitted by pushing buttons on handheld response devices. Following the submission of each decision, the market was updated. The final value of the portfolio determined the subject's compensation. The subject had no prior knowledge of these markets, nor had any direct experience in stock-market trading. Throughout performance of this task, we monitored dopamine release using fast-scan cyclic voltammetry at the carbon-fiber microelectrode assembly with a sampling rate of 10 Hz. We observed changes in the voltammetric signal that were consistent with fluctuations in dopamine concentration as indicated by linear regression analysis with a dopamine template obtained from the rat brain (e.g., Figure 1f inset). Furthermore, using principle component regression with a training set obtained in rats [19]–[21] we predicted the value of the voltammetric signal attributable to dopamine (Figure 1f). In a preliminary assessment, we tested for correlation between this signal and pertinent behavioral indices (Figure S1). A strong correlation was observed between the dopamine signal and the market value throughout the sequential-investment task (r^2^ = 0.549, p<0.000001). Furthermore, when these variables were normalized to their own standard deviations (z-scored), the linear regression slope was 0.91 indicating a near-unity relationship in these variables. Surprisingly, the slope of the dopamine signal over a period five seconds *prior* to a market price update correlated with subsequent market returns (r^2^ = 0.156, slope = 0.99, p = 0.0000482, n = 100 choices) demonstrating that it is a significant predictor of future market activity. To test the capacity of this prediction, we constructed a trading model based on the fluctuations in the dopamine signal leading up to the market price updates, which invested 100% (all in) when the dopamine slope was positive and 0% (all out) when the slope was negative. Over the 5 markets played, this trader model earned 202 points (a gain of 175%), more than two times the amount earned by the subject's expressed behavior. These data demonstrate that the information encoded in the dopamine signal of this patient is potentially useful for economic decision making.

We have demonstrated the use of existing electrochemical methods [8], [9], [10], [15], [19]–[21] – modified for compatibility with the human brain and existing neurosurgical equipment – to measure sub-second dopamine release in human striatum. It is important that such technology be safe for human subjects. Our modifications adapted the electrochemical probe to the DBS electrode implantation apparatus, thus the majority of the brain tissue affected by the procedure was not different from that of the typical surgical procedure. The tip of the carbon fiber probe is significantly smaller that the sharp tungsten electrodes used to functionally map the region and the DBS electrode that is left in place for therapeutic outcome. Measurements recorded during implantation demonstrated that the tip of the carbon fiber electrode remained intact during the implantation procedure as expected. From all accounts of the neurosurgeon and the surgical staff, the placement of the DBS electrode, its intra-operative efficacy, and post-operative efficacy were unaffected by our procedure. The patient recovered as expected and received therapeutic benefits from the DBS electrode well within the expected range of efficacy. To our knowledge the procedure had no adverse effects on the patients comfort or clinical outcome. The present work and the recently reported measurement of human substantia nigra neuron activity in Parkinson's disease patients [14] suggest that dopamine neuron activity and dopamine release can be investigated in humans with a depleted dopamine system.

In summary, we have demonstrated the first *in situ* measurements of dopamine release in a human brain; and have generated preliminary evidence that dopamine release can be tracked and investigated in the context of human decision-making behavior. This methodological demonstration opens the door to future investigations utilizing sub-second chemical measurements in the human brain, which should yield important insights into the role of dopamine signaling in human decision-making.

## Methods

All of the work presented here was performed under the approval of the University of Washington's Institutional Animal Care and Use Committee (rodent work: 4073-1) and Baylor College of Medicine's Institutional Review Board (human work: H-24018). All procedures conform to the ethical principles outlined in the Declaration of Helsinki. Baylor College of Medicine's Institution Review Board approved this study in humans and written informed consent was obtained from the patient. Detailed methods of all procedures are available in the supplementary online material at http://www.plosone.org.

## Supporting Information

Figure S1 **Dopamine release in the caudate tracks the market price during the sequential investment task.** (**a**) Sequential investment task. For each decision in the game the subject is presented three pieces of information: (1) market trace (red), (2) portfolio value (bottom left, “**139**” in this example) and (3) the most recent fractional change in portfolio value (bottom right, “**−23.92%**” in this example). The vertical grey bar (middle) is toggled by the subject to determine how much of one's own portfolio to invest in 10% increments (range = 0%–100%; “**50**” % is shown here). (**b**) (**left**) Representative market (magenta trace: normalized market value, N = 20 investment decisions) and corresponding dopamine measurement (black trace: normalized DA response in human caudate, 10 Hz sampling). Scale bar: normalized units (σ = 1 standard deviation) along the vertical axis and time (seconds) along the horizontal axis. Linear regression of the dopamine response on to the market price shows a significant correlation: p<0.000001; regression slope = 0.91; and r^2^ = 0.549, N = 100 decisions. (**middle**) Scatter plot of the dopamine slope (5 seconds prior to market update) and market returns along with the fitted regression line. (slope = 0.99; p = 0.0000482, r^2^ = 0.155, N = 100 decisions). (**right**) Bar plot comparing the performance of two agents playing the investment task: two agents are compared: patient “MH” and an agent modeled after the dopamine signal (DA). (**c**) Cyclic voltammograms are measured once every 100 ms in the human striatum. At each data point, a principal components regression based model (Heien et al., 2005) is used to derive the dopaminergic contribution to the measured signal. Here Q-values (Q_t_) provide a measure of the unaccounted residual variance once the PCR-model is applied. Instances where Q_t_ surpasses an experimentally determined Q-threshold (Q_σ_) are plotted here (“+Q_t_”, **magenta** data points) below the predicted dopamine (**black**) and known market value (**blue**) traces. By this analysis any dopamine data point accompanied by a magenta point is predicted from a cyclic voltammogram that has a significant amount of unaccounted for residual variance given the PCR model used.
(TIFF)

Methods S1 (DOC)
