# Supplementary figures and images for: Sub-Second Dopamine Detection in Human Striatum
Source: PLoS One. 2011 Aug 4;6(8):e23291. doi: 10.1371/journal.pone.0023291 (PMC3150430; doi:10.1371/journal.pone.0023291)

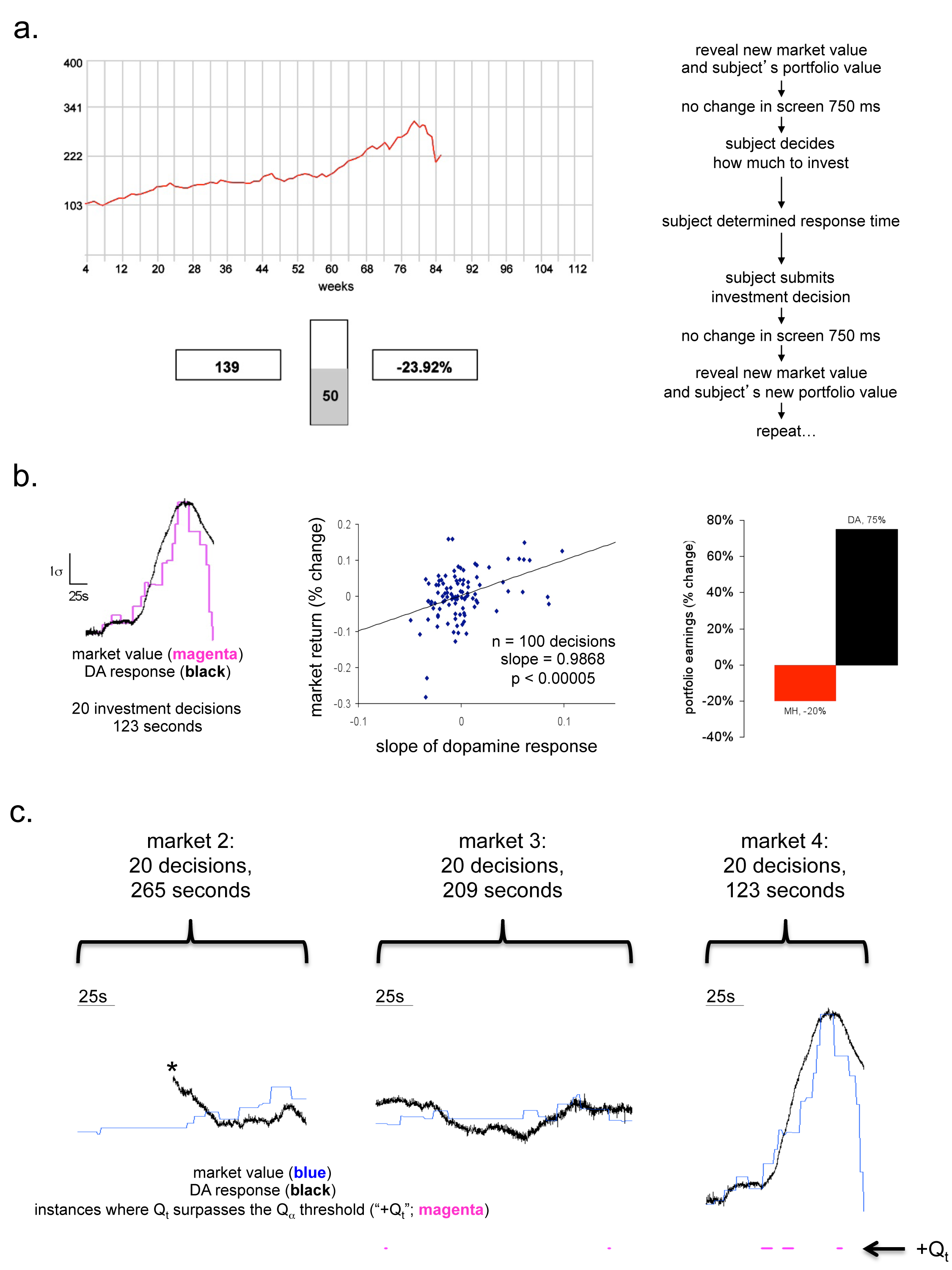

Supplement: Figure S1 — Dopamine release in the caudate tracks the market price during the sequential investment task. (a) Sequential investment task. For each decision in the game the subject is presented three pieces of information: (1) market trace (red), (2) portfolio value (bottom left, “139” in this example) and (3) the most recent fractional change in portfolio value (bottom right, “−23.92%” in this example). The vertical grey bar (middle) is toggled by the subject to determine how much of one's own portfolio to invest in 10% increments (range = 0%–100%; “50” % is shown here). (b) (left) Representative market (magenta trace: normalized market value, N = 20 investment decisions) and corresponding dopamine measurement (black trace: normalized DA response in human caudate, 10 Hz sampling). Scale bar: normalized units (σ = 1 standard deviation) along the vertical axis and time (seconds) along the horizontal axis. Linear regression of the dopamine response on to the market price shows a significant correlation: p<0.000001; regression slope = 0.91; and r2 = 0.549, N = 100 decisions. (middle) Scatter plot of the dopamine slope (5 seconds prior to market update) and market returns along with the fitted regression line. (slope = 0.99; p = 0.0000482, r2 = 0.155, N = 100 decisions). (right) Bar plot comparing the performance of two agents playing the investment task: two agents are compared: patient “MH” and an agent modeled after the dopamine signal (DA). (c) Cyclic voltammograms are measured once every 100 ms in the human striatum. At each data point, a principal components regression based model (Heien et al., 2005) is used to derive the dopaminergic contribution to the measured signal. Here Q-values (Qt) provide a measure of the unaccounted residual variance once the PCR-model is applied. Instances where Qt surpasses an experimentally determined Q-threshold (Qσ) are plotted here (“+Qt”, magenta data points) below the predicted dopamine (black) and known market value (blue) traces. By this [file pone.0023291.s001.tiff]
