# Supplementary material for: Sub-Second Dopamine Detection in Human Striatum
Source: PLoS One. 2011 Aug 4;6(8):e23291. doi: 10.1371/journal.pone.0023291 (PMC3150430; doi:10.1371/journal.pone.0023291)
Supplement: Methods S1 — (DOC) [file pone.0023291.s002.doc]

**Supplemental Online Methods**

**Electrode fabrication (glass-insulated electrodes):** A carbon fiber (7-μm diameter, Goodfellow Corporation) was inserted into a borosilicate-glass capillary (1.0-mm OD, 0.5-mm ID; A-M Systems). The capillary was pulled in an microelectrode puller (Sutter Instrument Company) to a tapered tip that forms a tight seal around the carbon fiber. A wire lead was inserted into the back end of the capillary and electrical connection was made to the carbon using silver paint.

**Electrochemistry:** We used fast-scan cyclic voltammetry (FSCV) to detect dopamine [2] by applying a triangular potential ramp (-0.425 to +1.380 V versus stainless steel, 400 Vs^-1^) to the carbon-fiber electrode assembly, 10 Hz. Currents associated with an analyte’s redox reactions occur at unique potentials (for dopamine, peak oxidation occurs at +0.65 to +0.75 V and reduction at -0.15 to -0.25 V). These current responses are characteristic of the electroactive analyte and are used for analyte identification when plotted against the corresponding applied potential to form a cyclic voltammogram. Potential ramp application, data acquisition and data analysis was computer controlled with PC-based system using two data acquisition cards (National Instruments PCI-6052E and PCI-6711) controlled with software written in LabVIEW (National Instruments).

All data collected in the surgical suite were collected with a mobile voltammetry data acquisition system (locally built). A voltammetry headstage, similar to that used in [2] but with extended cables was interfaced with data acquisition cards (National Instruments USB-6221 and USB-6251) that were housed in a metal case and connected to laptop computer via a USB 2.0 interface.

***In vitro* electrode characterization:** Electrode assemblies were inserted into a flowstream [8] (4 ml min^-1^) of artificial cerebrospinal fluid (154.7 mM Na^+^, 2.9 mM K^+^, 132.49 mM Cl^−^ and 1.1 mM Ca^2+^ at pH 7.4), where a bolus dose of known dopamine concentrations (62.5, 125, 250, 500 or 1000 nM) were introduced via a computer controlled injection valve system (Upchurch Scientific).

***In vivo* electrode characterization:** All procedures on animals were performed in compliance with University of Washington Institutional Animal and Use Committee approved protocol. Male Sprague-Dawley rats (350-400 g) were anesthetized with urethane (1.5 g kg^-1^). Body temperature was maintained with deltaphase heating pad (Braintree Scientific). Scalp was removed and connective tissue retracted to expose skull bone. Holes were drilled for stereotactic placement of reference electrode, two recording electrodes (1.3 mm anterior, 1.5 and 2.5 mm lateral of bregma and 4.5 to 5.0 mm ventral of cranial surface) and a bi-polar stimulating electrode (4.6 mm posterior, 1.3 mm lateral of bregma, Plastics One), which targeted the medial forebrain bundle. The stimulating electrode was lowered incrementally until optimal dopamine release was obtained. Dopamine release was elicited by applying an optically isolated, constant-current stimulus train of bi-phasic square wave pulses (pulse width: 4ms) at 60 Hz, 120 pulses, 300 µA.

**Targeting the caudate nucleus:** FHC microTargeting® system, Leksell stereotactic system®, and Medtronic Navigation® image guided targeting system were used to target the dorsal tier of the caudate nucleus in a patient undergoing DBS electrode implantation surgery. The sensor is retracted in the protective tube while the assembly was lowered through a stainless-steel guide tube and into the brain. The sensor is then extended into brain tissue once the protective tube is in the caudate nucleus. Thus the carbon fiber is protected during most of the electrode placement procedure. For patient MH, the subthalamic nucleus (STN) was the target for the DBS electrodes. During DBS electrode implantation, neurosurgeons must plan a trajectory that places the DBS electrodes in the target and in the preferred orientation within the target. Other decision criteria include but are not limited to avoiding blood vessels and other regions of the brain where known problems may arise if the electrodes were to pass through. These parameters are all accounted for by the neurosurgeon performing the procedure and are variable from patient to patient depending on the circumstances specific to each patient. In many DBS electrode implantation procedures the caudate can present itself as a structure that the DBS electrodes pass by en route to the STN. For this patient, the pre-operative planning revealed that the caudate could safely be targeted without compromising the trajectory of the DBS electrodes (this may not be the case for all patients). The caudate was chosen as the primary target for dopamine recordings since previously published results using fMRI and this task suggested that important dopamine related signals were elicited there [3,7].

The procedure (electrode placement, dopamine recording, and execution of the sequential investment task) added ~30 minutes to the procedure. This “added time” was well within the accepted range that the subject (and surgeon) could expect for a DBS electrode implantation procedure to be completed. For example, neurosurgeons will typically look for an optimal trajectory and functional response prior to placing the DBS electrodes. This procedure can take variable amounts of time, especially if more than two or three passes are required to get the functional response the neurosurgeon is looking for. Our protocol kept this in mind. If it took too long to get the optimal trajectory, then the experiment would be called off. Thus the patient was not exposed to any additional time of what is acceptable for the DBS electrode implantation procedure. This was explained to the patient prior to their enrollment in the study.

**Determination of dopamine:** We used linear regression [9] and principle component regression [4-6] to compare *in vivo* electrochemical signals to a training set from the rat brain generated from electrically evoked dopamine and pH, and electrode-background drift.

Linear regression: We used a method that is traditional to FSCV dopamine detection [9]: here a linear regression based match between the measured signal (black trace) and a template response (red trace) is used to identify events where dopamine contributes enough to the electrochemical signal such that the cyclic voltammogram can be classified as a dopaminergic event. The black traces in these insets would not be expected to be completely similar since they are minimally preprocessed (background subtracted) data collected at different points in time, thus the electrochemical signature may be altered by changes in the concentration of chemical species occurring between these two points in time. These traces are compared to the red traces (which are the same in both insets) using linear regression to quantify “goodness of match” between the traces acquired in the human (black) and those acquired in a typical rodent experiment (red) where dopamine release is stimulated under controlled circumstances. CVs were objectively compared with correlation analysis as previously described and found to match the dopamine template within the criteria of previous studies [9].

Principal component regression: We followed the principal component regression analysis methods previously described by Heien and colleagues [5,6]. A training data set was generated by varying the concentration of dopamine, pH, and measured electrode drift (n = 16 CVs). All training data were collected in rats as previously described [1,5,6,13]. We selected the first four eigenvectors following principal component analysis of the training data set to include in the principal component regression model. These four factors explain 95% of total variance in the training data set and were chosen using Malinowski’s F-test for rank determination according to Keithly et al [10].

**Sequential investment task (see Figure S1)**: The subject was endowed with $100 at the beginning of the sequential investment task. For each decision in the game, the subject must decide how much of their portfolio to invest (0%-100%, in 10% increments) based on three pieces of information presented at each decision point: (1) *market trace*, i.e., current market value (red trace, top of the screen), (2) the subject’s *current portfolio value* (bottom left of screen; in example screen shot “**139**”) and (3) the *most recent fractional change in the subject’s portfolio* value, i.e., gains or losses incurred following the last decision (bottom right of screen; in example screen shot “**-23.92%**”). A vertical bar (bottom middle; in example screen shot it is set at “**50**”, which is equivalent to 50%) is a selector bar that the subject toggles up and down to determine how much of one’s own portfolio to invest in the market; the bar is grey when inactive and becomes red when the subject is allowed to use it. Once the subject submits their answer, the screen holds for 750 ms before updating all three pieces of information. After the update, the screen holds for 750 ms and then the subject is allowed to make another decision. The pace of the subject determines the length of each decision point. This subject played 6 markets (20 decisions per market) while the electrochemical electrode was placed, and active, in the right caudate. The subject’s portfolio value at the end of the task determines the compensation received. The markets used in this task reflect actual historical stock markets where human decision-making determined the outcome of the market price. Near the end of market 1, the subject requested to be reinstructed; at the asterisk in Figure S1c, the subject finished receiving instructions and began to play independently; thus, all analyses are restricted to the data collected from markets 2-6 (100 decisions).

**Linear regression analyses**: We follow previous methods for determining the validity of attributing the presence of dopamine by linear regression of cyclic voltammograms collected in the human striatum to those collected in a more conventional paradigm (electrically evoked dopamine release from the rat striatum). One-to-one comparison of current from each point along the two voltammograms such that a linear plot and regression analyses quantifies the similarity between the two traces [9,11,12].

We also performed a linear regression using normalized values of the dopamine signal and variables derived from the market task. Normalization was performed by calculating the z-score of each variable such that variations across signals could be compared. For linear regression of the dopamine signal on to the market value, the dopamine level was re-sampled at time points when the market price fluctuated. In addition, we performed a time-series regression modeling to predict the market price from the fluctuations in the dopamine level at each market price change and a first order lag term as an additional dependent variable. In Figure S1b (middle) the slope of the dopamine signal was calculated by fitting a regression line through the values of the 10Hz sampled dopamine signal 5 seconds prior to a market price fluctuation. The slope of this line was then regressed on to the changes in market price.

**Dopamine model trader:** A simple model for trading behavior used the sign of the slope of the dopamine signal 5 seconds prior to the market change. If the slope was positive, the model invested 100% of the portfolio; if the slope was negative, the model invested 0% of the portfolio.

**Data analysis:** Voltammetric data were analyzed using software written in LabVIEW (National Instruments), and statistical analyses were carried out with Prism (GraphPad Software) and MATLAB (MathWorks, Inc).

**Supplemental Results**

With the electrode implanted, the subject engaged in a behavioral task [3] (Figure S1a) where the current value and recent history of a stock market was graphically represented on a laptop monitor. The subject chose the proportion of a portfolio (initially valued at $100) to be invested in the stock market. These decisions were submitted by pushing buttons on handheld response devices. Following the submission of each decision, the market was updated. The final value of the portfolio determined the subject’s compensation. The subject had no prior knowledge of these markets, nor had any direct experience in stock-market trading. The patient made 120 decisions over 6 distinct markets, but asked instructional questions during the first trials before engaging in the task without further instruction beginning in the second market. Thus, our primary analyses are restricted to the last 5 markets (100 decisions). During this period, the subject’s portfolio changed from $115 to $92, a 20% loss (Figure S1b, right panel, black bar). We determined that the rate and timing of button presses did not account for the observed fluctuations in dopamine delivery. However, a strong correlation was observed between the normalized (z-scored) dopamine signal and market value throughout the sequential-investment task (r^2^ = 0.549, p < 0.000001, N = 100 decisions; Figure S1b left panel). The linear regression slope was 0.91 indicating a near-unity relationship in these variables. We also assessed whether autocorrelation in the dopamine levels were a significant predictor in a time-series regression, but found that this additional dependent term did not significantly change the interpretation of the relationship between the market response and the dopamine levels; the p-value for this correlation changed from p<0.000001 to p<0.001, while no significance was found for the lag variable (p = 0.43) in explaining the market returns. Surprisingly, the slope of the dopamine signal over a period five seconds just prior to a market price update correlated with subsequent market returns (Figure S1b, middle panel: r^2^ = 0.156, slope = 0.99, p = 0.0000482, N = 100 decisions) demonstrating that the dopamine signal is a significant predictor of future market activity. To test the capacity of this prediction, we constructed a trading model based on the fluctuations in the dopamine signal leading up to the market price updates. This model invested 100% (all in) when the dopamine slope was positive and 0% (all out) when the slope was negative. Over the 5 markets played (100 decisions), the model earned 202 points (a gain of 175%), more than two times the amount earned by the subject’s expressed behavior (Figure S1c, right panel). These behavioral data and associated dopaminergic measurements are preliminary and in need of replication; however, these preliminary results suggest that information encoded in the dopaminergic signals in human brains are potentially useful for economic decision-making.

**References**

1. Phillips PE, Stuber GD, Heien ML, Wightman RM, Carelli RM. (2003) Subsecond dopamine release promotes cocaine seeking. Nature 422(6932): 614-618.

2. Clark JJ, Sandberg SG, Wanat MJ, Gan JO, Horne EA, et al. (2010) Chronic microsensors for longitudinal, subsecond dopamine detection in behaving animals. Nat Methods 7(2): 126-129.

3. Lohrenz T, McCabe K, Camerer CF, Montague PR. (2007) Neural signature of fictive learning signals in a sequential investment task. Proc Natl Acad Sci U S A 104(22): 9493-9498.

4. Kramer R. (1998) Chemometric techniques for quantitative analysis. New York: Marcel Dekker.

5. Heien ML, Johnson MA, Wightman RM. (2004) Resolving neurotransmitters detected by fast-scan cyclic voltammetry. Anal Chem 76(19): 5697-5704.

6. Heien ML, Khan AS, Ariansen JL, Cheer JF, Phillips PE, et al. (2005) Real-time measurement of dopamine fluctuations after cocaine in the brain of behaving rats. Proc Natl Acad Sci U S A 102(29): 10023-10028.

7. Chiu PH, Lohrenz TM, Montague PR. (2008) Smokers' brains compute, but ignore, a fictive error signal in a sequential investment task. Nat Neurosci 11(4): 514-520.

8. Kristensen EW, Wilson RL, Wightman RM. (1986) Dispersion in flow injection analysis measured with microvoltammetric electrodes. Anal Chem 54: 986-988.

9. Cheer JF, Wassum KM, Heien ML, Phillips PE, Wightman RM. (2004) Cannabinoids enhance subsecond dopamine release in the nucleus accumbens of awake rats. J Neurosci 24(18): 4393-4400.

10. Keithley RB, Carelli RM, Wightman RM. (2010) Rank estimation and the multivariate analysis of in vivo fast-scan cyclic voltammetric data. Anal Chem 82(13): 5541-5551.

11. Roitman MF, Stuber GD, Phillips PE, Wightman RM, Carelli RM. (2004) Dopamine Operates as a Subsecond Modulator of Food Seeking. J Neurosci 24(6): 1265-1271.

12. Stuber GD, Roitman MF, Phillips PE, Carellie RM, Wightman RM. (2005) Rapid Dopamine Signaling in the Nucleus Accumbens during Contingent and Noncontingent Cocaine Administration. Neuropsychopharmacology 30: 853-863.

13. Heien ML, Khan AS, Ariansen JL, Cheer JF, Phillips PE, et al. (2005) Real-time measurement of dopamine fluctuations after cocaine in the brain of behaving rats. Proc Natl Acad Sci U S A 102(29): 10023-10028.
